# Empirically Estimated Electron Lifetimes in the Earth's Radiation Belts: Van Allen Probe Observations

**DOI:** 10.1029/2019GL086053

**Published:** 2020-02-07

**Authors:** S. G. Claudepierre, Q. Ma, J. Bortnik, T. P. O'Brien, J. F. Fennell, J. B. Blake

**Affiliations:** ^1^ Space Sciences Department The Aerospace Corporation El Segundo CA USA; ^2^ Department of Atmospheric and Oceanic Sciences University of California Los Angeles CA USA; ^3^ Center for Space Physics Boston University Boston MA USA

**Keywords:** radiation belt, lifetime, decay, pitch angle diffusion, loss, wave particle interaction

## Abstract

We use measurements from NASA's Van Allen Probes to calculate the decay time constants for electrons over a wide range of energies (30 keV to 4 MeV) and L values ( L = 1.3–6.0) in the Earth's radiation belts. Using an automated routine to identify flux decay events, we construct a large database of lifetimes for near‐equatorially mirroring electrons over a 5‐year interval. We provide the first accurate estimates of the long decay timescales in the inner zone ( ∼100 days), which are highly resolved in energy and free from proton contamination. In the slot region and outer zone, we compare our lifetime calculations with prior empirical estimates and find good quantitative agreement (lifetimes ∼1–20 days). The comparisons suggest that some prior estimates may overestimate electron lifetimes between L≈ 2.5–4.5 due to instrumental effects and/or background contamination. Previously reported two‐stage decays are explicitly demonstrated to be a consequence of using integral fluxes.

## Key Points

- A large database of radiation belt electron decay timescales is calculated from Van Allen Probes MagEIS measurements
- We provide the first accurate estimates of these timescales over a wide range of energies in the inner zone, free from proton contamination
- Outer zone decay timescales generally agree well with prior estimates; some differences exist and may be due to instrumental effects

## 1 Introduction

The Earth's electron radiation belts rarely, if ever, reach a state of equilibrium and exist in a constant state of flux, the result of competition between various source and loss processes. During highly dynamic intervals, such as geomagnetic storms, both the source and loss processes generally operate on fast timescales ( ∼1 day or less). Outside of storm times, the balance of longer‐timescale processes (e.g., pitch angle and radial diffusion) determines the overall configuration of the belts, which are often observed to decay exponentially following enhancements. Many authors have calculated these electron decay time constants, or “lifetimes,” from observations at various energies and locations throughout the belts to help elucidate the relevant physics (e.g., Albert, 2000; Baker et al., 2007; Benck et al., 2010; Fennell et al., 2012; Meredith et al., 2006, 2009; Ripoll et al., 2015; Roberts, 1969; Seki et al., 2005; Su et al., 2012; Vampola, 1971; West Jr et al., 1981). Such lifetime estimates are useful for radiation belt modeling, whereby the complexity of the problem can be reduced by incorporating all of the loss processes and loss physics into a single model parameter. Accurate calculations of electron lifetimes are also important for quantitative assessments of the radiation hazards posed to spacecraft, particularly in the inner zone where relativistic electrons appear sporadically and exhibit long lifetimes. This manuscript seeks to obtain accurate estimates of these lifetimes from observations and compare them with prior empirical estimates, when and where such comparisons are possible (e.g., there are no prior estimates in the inner zone with which we can compare, and very few estimates at energies <200 keV throughout the belts). A companion paper uses the lifetime estimates to constrain and inform our understanding of the relevant physical processes that contribute to the loss of electrons from the radiation belts.

## 2 Data and Methods

The primary data used in this work are measurements from the Magnetic Electron Ion Spectrometer (MagEIS; Blake et al., 2013) sensors aboard NASA's Van Allen Probes (Mauk et al., 2013). The MagEIS electron spectrometers measure the angular distribution over the spacecraft spin period ( ∼11 s) for electrons in the energy range ∼30 keV to ∼4 MeV. The electron fluxes are presented here as daily averages in fixed L bins (0.1 L‐width) with McIlwain L obtained from the Olson and Pfitzer (1977) quiet model. The measurements are extracted near the magnetic equator when $B/B_{eq}\leq 1.1$, where $B/B_{eq}$ is the ratio of the magnetic field strength at the spacecraft to that at the magnetic equator (both obtained from the model). The fluxes are averaged between 80° and 100° local pitch angle, which, for this $B/B_{eq}$ range, corresponds to equatorial pitch angles between 70° and 110°. Background‐corrected data (Claudepierre et al., 2015) are used exclusively, where the modified technique of Claudepierre et al. (2019) is employed. We present data from Probe B over the 5‐year interval from 1 April 2013 to 31 March 2018.

**Figure 1:**
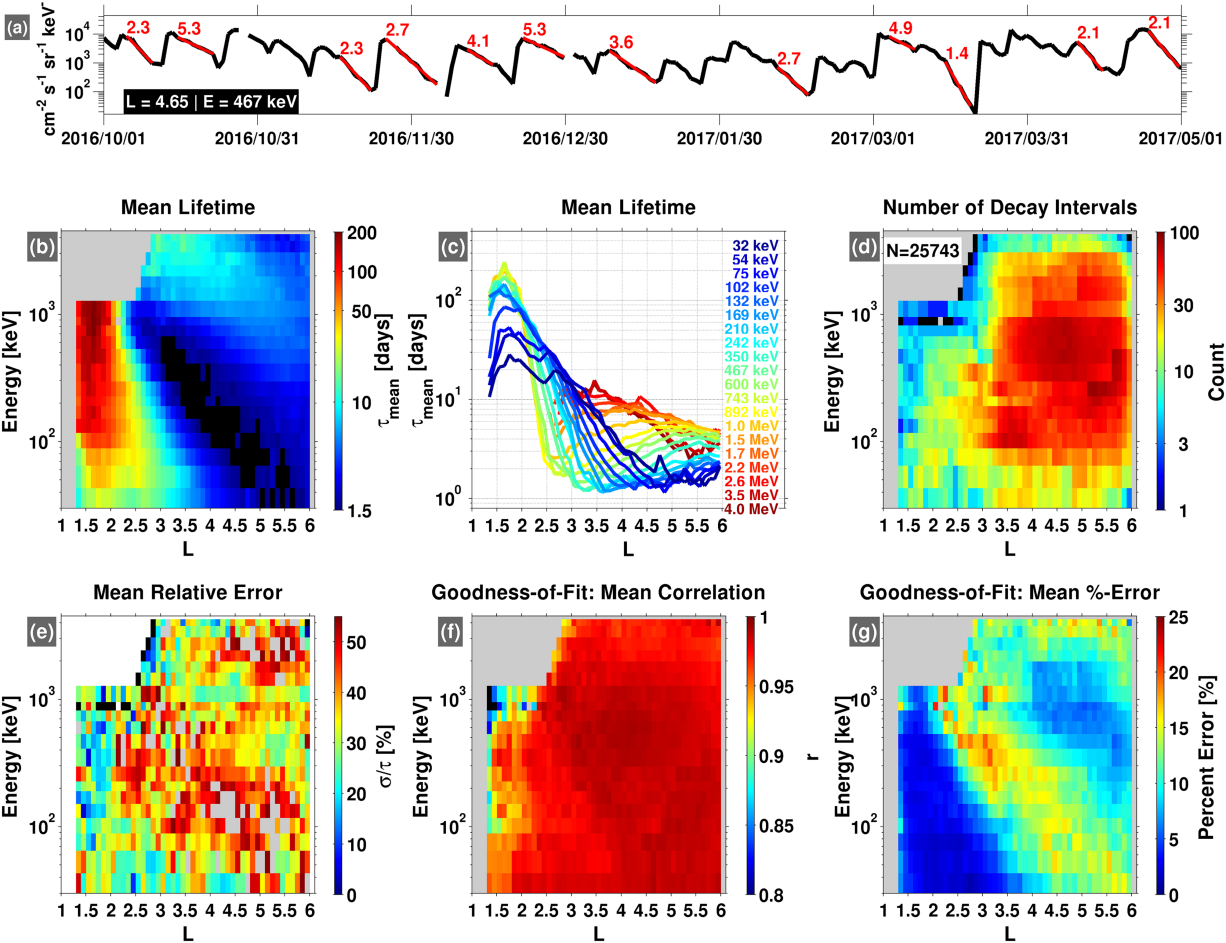
Summary of the decay timescales obtained from the automated algorithm. (a) Daily‐averaged, differential flux at L=4.65 for 467‐keV electrons. Exponential decays identified by the automated algorithm are highlighted in red with the calculated decay (e‐folding) times indicated, in days. (b) Mean lifetimes calculated in each energy and L bin (0.1 L‐width). (c) Same as panel (b) but here displayed in a line plot format. (d) The number of decay intervals identified in each energy and L bin, with the total number in all bins indicated ( N). (e) The mean relative error (standard deviation of the lifetimes divided by the mean lifetime) in each energy and L bin (note that gray color in this panel indicates a value above the maximum of the color scale). (f and g) Goodness‐of‐fit metrics in each energy and L bin, displaying the mean linear correlation coefficient ( r) and the mean percent error between the exponential fit and the flux. In all of the color panels, a black color indicates a value below the indicated color scale.

An automated algorithm has been developed to identify exponential decays and calculate the e‐folding times of the decays from the MagEIS electron measurements. This algorithm, which is described in greater detail in the supporting information, is based on the technique of Benck et al. (2010), which was in turn adapted from that of Meredith et al. (2006). The algorithm is designed to estimate decay times over time intervals where the fluxes are decreasing for at least 5 days. The flux time series are fit with an exponential function, $J(t)=J_{0}\;\exp(-t/\tau )$, using two goodness‐of‐fit parameters to ensure high‐quality fits, the linear correlation coefficient, and the percent error between the fit and the flux. We note that these parameters are L‐ and energy‐dependent, as is the total length of the time interval that is fit (see the supporting information). The fits are obtained at all L = 1–6, and we do not sort the decay timescales with respect to the plasmapause location, primarily because none of the prior works with which we compare have done so. In addition, since we obtain fits in fixed L bins, it is difficult to assign an “inside” or “outside” of the plasmasphere designation to an individual decay event, since the plasmapause could move across the fixed L bin during the decay interval. Figure 1a provides an example of the application of the automated procedure at L=4.65 and 467‐keV energy.

## 3 Results

Figure 1 presents statistical results from the decay timescale database obtained. Figure 1b shows the mean decay timescale, or “lifetime,” in each L and energy bin. Throughout this work, we use the terms decay timescale and lifetime interchangeably, noting that a more appropriate terminology is “effective” or “apparent” lifetime (e.g., Cunningham et al., 2018). The particle lifetime is an aggregate quantity that may include effects due to a number of different loss mechanisms, such as de‐energization due to nonlinear effects, and also tries to capture the lifetimes of a number of modes of the distribution simultaneously. Moreover, the calculated lifetimes could potentially be influenced by a source (e.g., inward radial transport from higher L) and thus may not always be representative of the true, underlying decay timescale. Thus, our calculated decay times represent upper bounds; the true decay times could be lower than those calculated if there are also source processes acting during the decay interval. It is a difficult task to exclude any inward transport or other source processes that may occur simultaneously with the decay, and we have not attempted to do so here. We emphasize that magnetopause shadowing events are effectively excluded from our database by the criteria above that the decay interval must be 5 days or longer. We have not attempted to remove very rapid local loss processes (e.g., microbursts) from the database, which will be smoothed out through the use of daily‐averaged fluxes.

Returning to Figure 1, we note that the fits are not constructed at L<1.3 due the fact that the fluxes are noisy and subject to considerable orbital effects in this region, from which generally poor results are obtained. In addition, fits are not performed at L>6 due to the large variability in the fluxes and the spatial coverage of the Van Allen Probes, which do not sample this region uniformly in time. The region of no data at high energy and low L in panel (b) is due to the fact that there have not been injections of >1.5‐MeV electrons into the inner zone at detectable levels during the Van Allen Probes era (Claudepierre et al., 2019; Fennell et al., 2015). Panel (c) shows the same data as panel (b) but presented in line plot format with each energy channel represented by a different color.

The profiles in panels (b) and (c) show that long electron lifetimes ( τ>100 d) are generally observed in the inner zone ( L<2.5) above 100 keV and below 1 MeV. In this energy range, the lifetimes peak near L=1.7, are largely independent of energy, and exhibit steep gradients on either side of the peak where the lifetime changes by ∼100 days in approximately one L shell ( dτ/dL not shown here). Moving outward in L, the lifetimes in this energy range then decrease rapidly toward the slot region, which can be identified in panel (b) as the deep blue and black region between L=3−5. Here, the lifetimes are on the order of 1–2 days, beyond which they increase slightly toward the outer region near L=6. At energies greater than 1 MeV, the lifetime profiles show a somewhat different character, with less radial dependence and values in the 5‐ to 10‐day range throughout the outer zone. As we detail in the companion paper, the general structure of the lifetime profiles as a function of energy and L is consistent with quasilinear pitch angle diffusion by various scattering mechanisms, such as Coulomb collisions (e.g., Cunningham et al., 2018) and VLF transmitters (e.g., Ma et al., 2017; Ross et al., 2019) for long lifetime effects and hiss/EMIC waves for shorter lifetime effects (e.g., below ∼1 MeV for hiss (e.g., Ripoll et al., 2019) and above ∼2 MeV for EMIC (e.g., Kersten et al., 2014)).

Figures 1d–1g display several parameters related to the statistical database and the automated algorithm. Figure 1d shows the total number of decay intervals identified in each ( L,E)‐bin. We see that the statistics are generally good at L>3.5 and for energies between 100 keV and 1 MeV, with fewer events in the inner zone and in the outer zone at higher energy. Figure 1e shows the mean relative error, which is defined as the standard deviation of the inferred lifetimes divided by the mean lifetime, expressed as a percentage. For example, in a given ( L,E)‐bin, if the mean lifetime is 10 days and the standard deviation is 1 day, then the mean relative error is 10%. We see that the mean relative error is generally less than 50%, that is, that the lifetimes inferred from the measurements vary by a factor of ∼2 around the mean, which is consistent with similar prior calculations (e.g., Baker et al., 2007; Benck et al., 2010). Figures 1f and 1g show the two goodness‐of‐fit criteria used in the automated search (see the supporting information), displaying high correlations and low errors between the fits and the fluxes throughout most of the region. As noted above and in the supporting information, the criteria on the quality of the fits must be relaxed in the inner zone and at the highest energies to boost statistics. The largest percent errors are observed in the slot region, due to the low flux levels often found there, which leads to enhanced Poisson (counting statistics) noise relative to other regions where higher flux levels are typically observed.

## 4 Discussion

**Figure 2:**
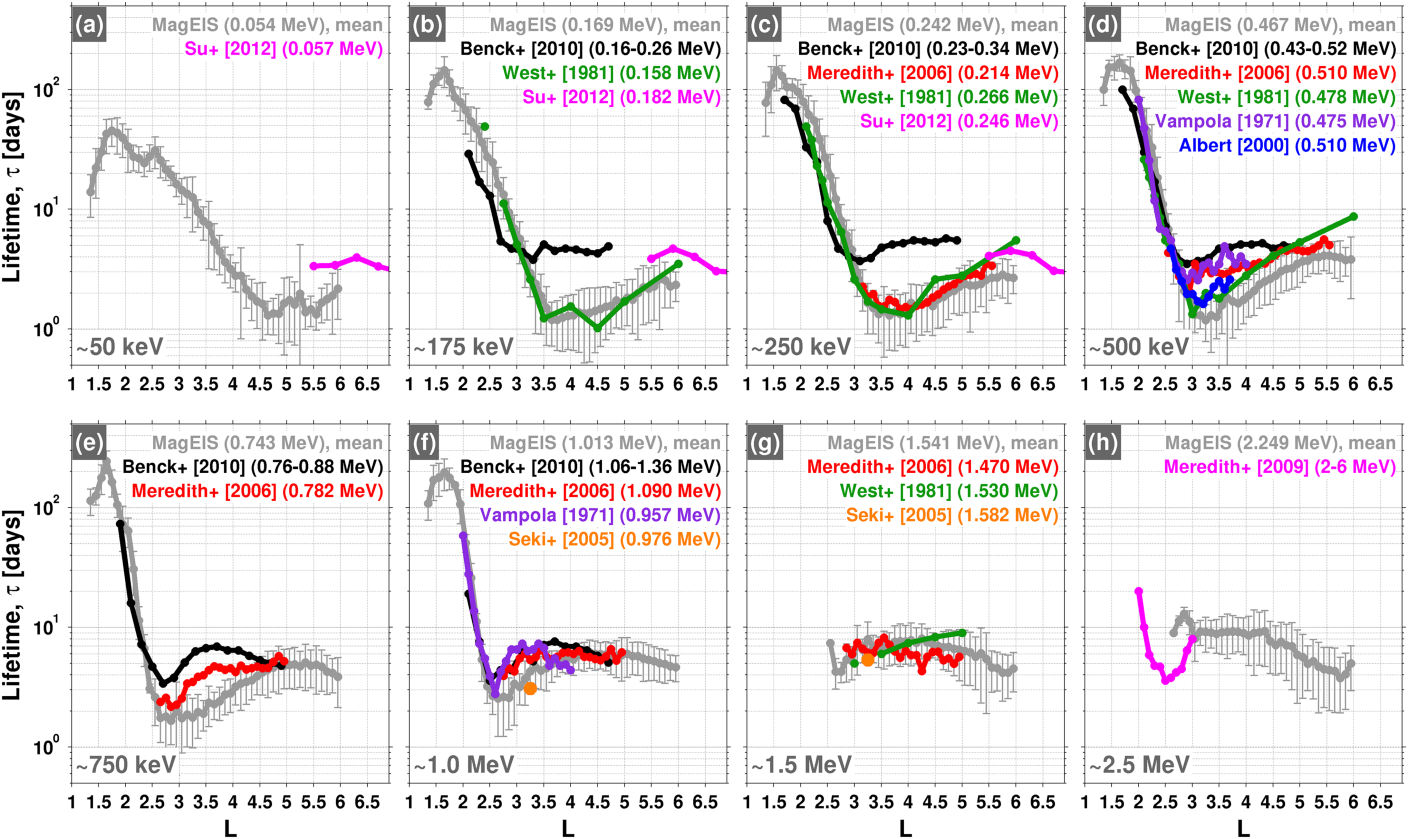
(a–h) Comparison of the mean lifetimes obtained from MagEIS (gray) with several previously published estimates (color) at eight fixed energies spanning ∼50 keV to ∼2.5 MeV. The error bars on the MagEIS lifetimes are one standard deviation on the mean.

Figure 2 compares the mean lifetimes from MagEIS with those obtained in previous works using similar techniques. In each panel, the MagEIS mean lifetime is shown in gray as a function of L at a fixed energy. Previously published lifetime estimates are displayed using different colors when those estimates are available in a comparable energy channel to the MagEIS channel. For example, Figure 2c shows an L profile of the mean lifetime estimate from MagEIS, along with four additional L profiles where the reference and energy channel are provided in the figure legend. The supporting information gives further details for each of the previously published estimates (spacecraft, instrument, orbit, etc.). Overall, we find good quantitative agreement between the MagEIS lifetime estimates obtained here and those from past works, specifically the outer zone lifetimes and the steep negative gradients in the slot region. Some discrepancies are noted, in particular that the MagEIS estimates tend to be slightly lower than some of the others. These differences are discussed in greater detail in the next sections, though we emphasize that there are no prior estimates in the inner zone in overlapping L regions ( L>1.3) with which we can compare, and very few at energies <200 keV in the slot and outer zone (see the supporting information). There were several estimates of inner zone electron lifetimes made following high‐altitude nuclear detonations in the late 1950s and early 1960s (Roberts, 1969). However, those were all obtained using integral sensors (e.g., those that measure the flux above some threshold energy), rather than the highly resolved differential channels used here, and we demonstrate below that lifetimes calculated from integral sensors are difficult to properly interpret and can be misleading.

### 4.1 Comparisons With Prior Estimates at Low Energy (50–250 keV)

At energies between 50 and 250 keV, the comparisons with the prior empirical estimates shown in Figures 2a–2c exhibit some quantitative disagreement with the MagEIS estimates. For example, the estimates from Su et al. (2012) are typically a factor of ∼2 greater than the MagEIS calculations and exceed the 1 σ error bar on the MagEIS estimates. We note, however, that these differences are at the highest Ls and lowest energies, where the MagEIS uncertainties are the largest (e.g., Figures 1d, 1e, and 1g). More noteworthy are the differences between the Benck et al. (2010) estimates and the others available for comparison. At 175 and 250 keV (Figures 2b and 2c) above L≈ 3, the Benck et al. (2010) estimates are noticeably out of family with the other estimates presented. The Benck et al. (2010) estimates are also higher than MagEIS at all other energies available for comparison, 160–1,360 keV (not shown here). Some of these discrepancies between the Benck et al. (2010) estimates and the others may be due to instrumental effects in the DEMETER/IDP measurements that were used (note that SAC‐C/ICARE measurements were also used in Benck et al., 2010). In particular, Selesnick et al. (2019) show that higher energy electrons reported by DEMETER/IDP may actually be measurements of lower‐energy electrons, due to the instrumental effect of pileup. In addition, they show that both pileup and deadtime lead to an artificially reduced flux at lower energies ( ≲200 keV). Without a more detailed analysis, it is difficult to ascertain how these two effects influence the lifetime estimates obtained from DEMETER measurements. Contamination from penetrating high‐energy electrons and/or bremsstrahlung may also contribute, as described below.

### 4.2 Comparisons With Prior Estimates at High Energy (0.5–4 MeV)

The comparisons with previous estimates shown in Figure 2 at energies >500 keV (Figures 2d–2h) generally show good quantitative agreement. The steep negative gradients in the lifetimes in the slot region are quantitatively consistent across all energies for all of the estimates, with respect to both the slope and the overall magnitude. However, between L≈ 2.5–4.5 some differences are noted between the MagEIS estimates and some of the prior works. For example, at 750 keV (Figure 2e) we see that both the Benck et al. (2010) and the Meredith et al. (2006) estimates are higher than the MagEIS lifetimes, exceeding the 1 σ error bars on MagEIS. In addition, the shape of the L profiles are different, with the Benck et al. (2010) and Meredith et al. (2006) lifetimes displaying a local maximum near L∼ 3.5, whereas the MagEIS lifetimes are increasing with L through this region. We now consider the possibility that some of these earlier estimates may be influenced by high‐energy electron contamination in this region.

Bremsstrahlung X‐rays are produced when high‐energy (e.g., multi‐MeV) electrons interact with the spacecraft and instrument materials. These x‐rays can register as counts in space‐based detectors that are designed to measure energetic particles (e.g., silicon solid state detectors). The MagEIS instrument was designed so that background contamination from bremsstrahlung X‐rays (and other sources) could be quantified and removed from the measurements, providing a highly robust measure of foreground electrons. We exploit this capability to examine how high‐energy electron contamination may influence lifetime calculations like those presented here.

**Figure 3:**
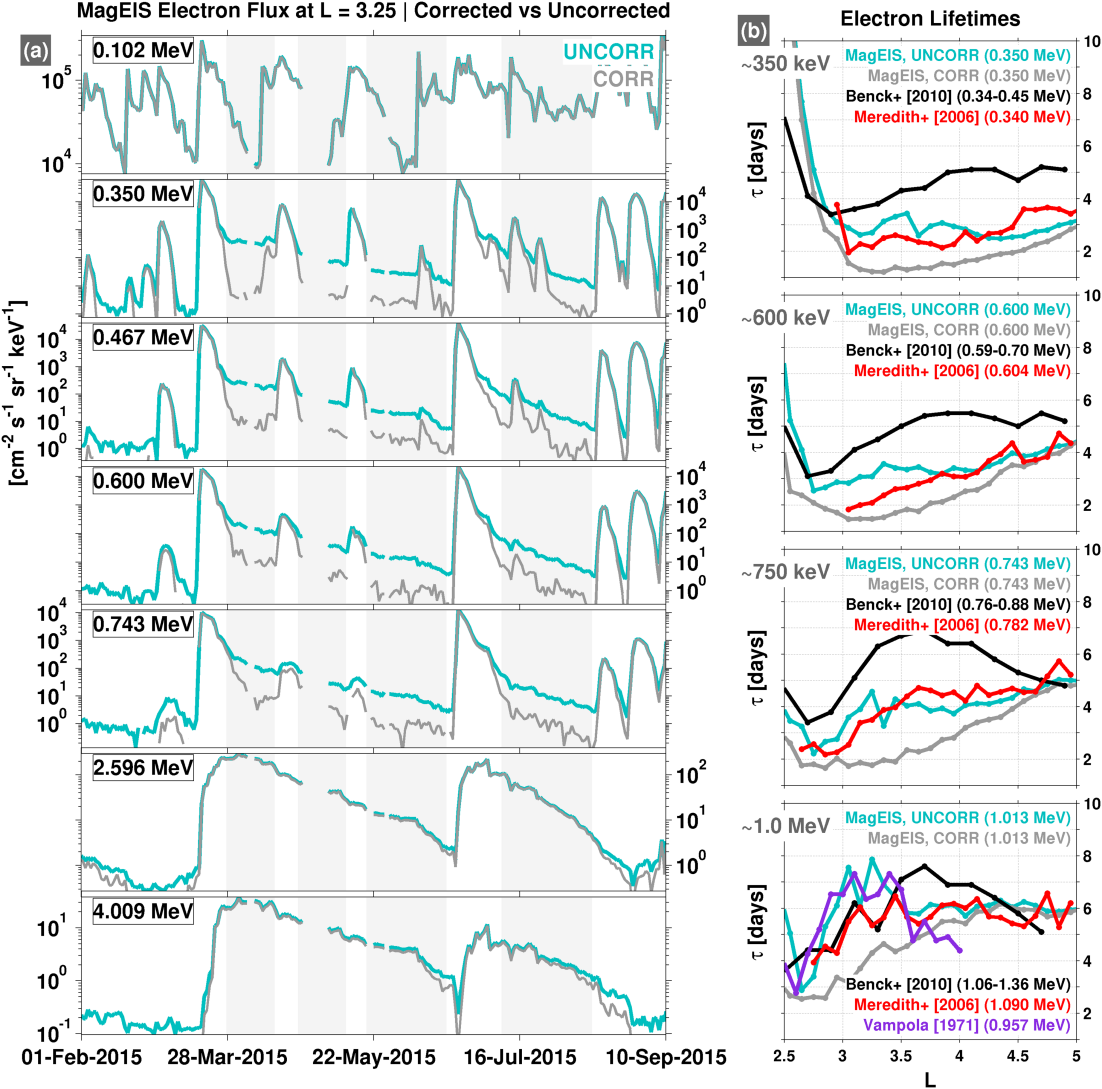
(a) A comparison of uncorrected (UNCORR) and background‐corrected (CORR) MagEIS electron flux at L = 3.25 for the indicated energy channels. Four time intervals are highlighted (gray shaded regions) as times during which bremsstrahlung from multi‐MeV electrons contaminates the uncorrected fluxes (most notably between 350 and 743 keV). (b) A comparison of lifetimes obtained from both the uncorrected and background‐corrected MagEIS electron fluxes, along with several previous empirical estimates.

Figure 3a shows time series of MagEIS electron flux measurements at L = 3.25 over the 100 keV to 4 MeV energy range. Both background‐corrected and uncorrected profiles are shown. During this time interval, we note that there are two strong enhancements of multi‐MeV (2.5–4.0 MeV) electrons, one in March 2015 and another in June 2015. Following these enhancements, the electrons at these energies decay slowly with a decay timescale on the order of 10 days. Note the influence that contamination from these multi‐MeV electrons has on the uncorrected flux profiles at lower energy. For example at 467 keV, during the time intervals highlighted with gray shading, the background‐corrected data reveal that the true dynamics are not that of steady, exponential decays, as is suggested by the uncorrected data. The bremsstrahlung contamination in the uncorrected data produces flux profiles that appear to decay exponentially, but this is simply a manifestation of the decay timescales of the multi‐MeV electrons that produce the bremsstrahlung. A similar effect is seen in the other energy channels between 350 and 743 keV.

We have performed an identical statistical analysis of electron lifetimes as described above (e.g., section 2), but now using the uncorrected MagEIS data. Figure 3b shows the results, where data from four of the same panels from Figure 2 are shown, but which now also include the calculations for the uncorrected MagEIS data. The effect that the bremsstrahlung contamination described above has on the lifetime calculations is clear. For example, between L∼ 3–5, bremsstrahlung contamination artificially increases the lifetime estimates obtained from the uncorrected MagEIS data, relative to the corrected data. This suggests that some prior lifetime estimates may be influenced by high‐energy electron contamination in this region. Specifically, note that the lifetimes obtained from the uncorrected MagEIS data are closer to the values obtained in prior works shown, in terms of both shape and magnitude. We note that this bremsstrahlung contamination interpretation is consistent with our knowledge of the MEA sensor on CRRES, which was used in the Meredith et al. (2006) study. CRRES/MEA shares a design heritage with MagEIS, employing a similar measurement technique, albeit with thicker detectors that are more susceptible to bremsstrahlung contamination than those used in MagEIS. Unfortunately, the telemetry requirements on the CRRES mission precluded downlinking the necessary data to perform background corrections on the MEA measurements. The results presented suggest that the Benck et al. (2010) estimates may similarly be contaminated by high‐energy electrons, in addition to the pileup and deadtime effects noted above.

### 4.3 Integral Flux Measurements and Two‐Stage Decays

As a final application of the capabilities and techniques presented here, we demonstrate the importance of carefully distinguishing between decay rates obtained from differential versus integral fluxes. Several past empirical lifetime estimates have been made using measurements from integral sensors, rather than the differential fluxes used here, most notably those made following high‐altitude nuclear detonations in the late 1950s and early 1960s (e.g., Roberts, 1969, and references therein). As noted by Fennell et al. (2012), electron flux decays observed by integral sensors often exhibit a two‐timescale or “two‐stage” decay, where a rapid initial decay ( τ∼ 1 day) is followed by a more gradual, slower decay ( τ∼ 20 days). Ripoll et al. (2015) argued that such observations are the consequence of the wide energy response of integral sensors combined with the wide range of decay timescales as a function of energy at a given L (e.g., Figure 1). We explicitly corroborate this assertion by exploiting the high‐energy resolution afforded by the MagEIS sensor, in conjunction with the techniques presented above.

**Figure 4:**
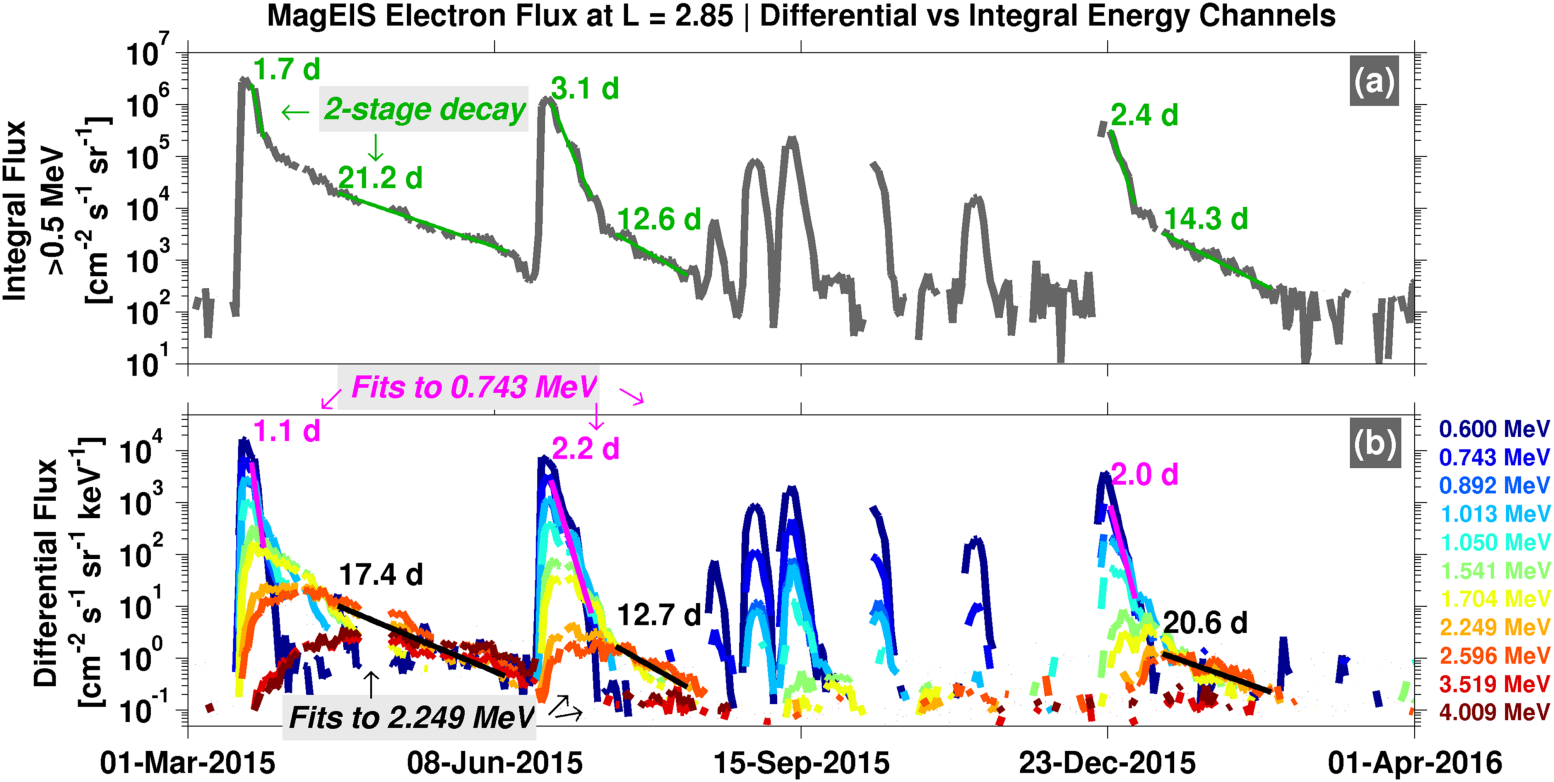
(a) MagEIS integral flux ( >0.5 MeV) at L = 2.85 over a 1‐year interval. Note the three instances of two‐stage decays (green), where an initial rapid decay ( τ≈ 1.7–3.1 days) is followed by a more gradual decay ( τ≈ 13–21 days). (b) MagEIS differential flux demonstrating that the two‐stage decays in the integral flux are due to energy‐dependent decay timescales.

Figure 4 compares MagEIS integral fluxes ( >0.5 MeV) in panel (a) with differential fluxes in panel (b) from the same energy range (0.5–4 MeV), at L = 2.85. Note that in Figure 1, the decay timescales at this L vary widely in this energy range, from ∼1 day at the lower energies to ∼10–20 days at the higher energies. In Figure 4a, we see that the integral fluxes are characterized by a two‐stage decay: a rapid, initial decay, followed by a slower decay as time progresses. The differential fluxes in Figure 4b reveal that the rapid initial decay in the integral flux is strongly influenced by the fluxes at the lower energy end of the integral channel ( ∼0.5 MeV) while the second, slower stage of the decay is dominated by the higher energy fluxes, which decay much more slowly than the lower energy fluxes. Thus, we urge caution when interpreting decay timescales obtained from integral channels, or even wide differential channels, since they can mix energy‐dependent decay rates, which we have demonstrated are a strong function of energy at a given L. In the companion paper, we show that the fastest energy‐dependent decay rates at a given L can be the result of multiple scattering mechanisms operating simultaneously, for example, hiss wave scattering at low energy (e.g., Ripoll et al., 2019) and EMIC wave scattering at high energy (e.g., Kersten et al., 2014).

## 5 Summary

We provide a comprehensive, long‐term (5‐year) database of energetic and relativistic electron decay timescales observed throughout the radiation belt region. This is the first such database obtained in a near‐equatorial orbit from a single sensor with high‐angular and energy resolution and quantifiable background rejection, which allows us to make the first truly quantitative such calculations in the inner zone. We find long lifetimes in the inner zone, short lifetimes in the slot region, and energy‐dependent lifetimes in the outer zone indicative of different loss mechanisms. These decay timescales obtained from MagEIS are largely consistent with previously obtained empirical estimates, when and where such estimates are available. We use the techniques presented to demonstrate that some prior estimates may be influenced by background contamination and that previously reported two‐stage decays are likely due to the use of wide energy (integral) flux measurements. A companion paper utilizes this database further to explore the physical mechanisms responsible for the observed decay timescales, which ultimately produce the quiet time structure of the radiation belts.

## Supporting information



Supporting Information S1
