# Supplementary material for: Empirically Estimated Electron Lifetimes in the Earth's Radiation Belts: Van Allen Probe Observations
Source: Geophys Res Lett. 2020 Feb 7;47(3):e2019GL086053. doi: 10.1029/2019GL086053 (PMC7375131; doi:10.1029/2019GL086053)
Supplement: Supplementary file 1 — Supporting Information S1 [file GRL-47-e2019GL086053-s001.pdf]

Supporting Information for "Empirically estimated electron lifetimes in the Earth's radiation belts: 1. Observations"

S. G. Claudepierre[1,2], Q. Ma[2,3], J. Bortnik[2], T. P. O'Brien[1], J. F. Fennell[1], and J. B. Blake[1]

[1]Space Sciences Department, The Aerospace Corporation, El Segundo, California, USA

[2]Department of Atmospheric and Oceanic Sciences, UCLA, Los Angeles, California, USA

[3]Center for Space Physics, Boston University, Boston, Massachusetts, USA

Contents of this file

1. Text S1 to S3
2. Figures S1 to S4
3. Table S1

Introduction

The following information is provided in support of the main manuscript. We provide further details on the empirical lifetime estimates from prior published works that are used in the main manuscript, including the spacecraft, orbit, and instrumentation, with all acronyms spelled out. We also provide additional details regarding the automated algorithm that is used to obtain the empirical lifetime database.

Text S1.

Table S1 lists the details on the empirical lifetime estimates from prior published works that are used in the main manuscript. The following sources have been used, with all of the orbital parameters obtained from the NASA Space Science Data Coordinated Archive (https://nssdc.gsfc.nasa.gov):

Vampola (1971) used measurements from the Magnetic Electron Spectrometer (MES) instrument on Orbiting Vehicle 3, number 3 (OV3-3). OV3-3 operated in a Highly Eccentric Orbit (HEO) with launch parameters of 358 km, 4479 km, 81.47° (perigee, apogee, and inclination, respectively).

West, Buck, and Davidson (1981) used measurements from the Electron and Proton Spectrometer (EPS) instrument on Orbiting Geophysical Observatories, number 5 (OGO5). OGO5 operated in a HEO orbit with launch parameters of 272 km x 23 R_E, 31.1° (perigee, apogee, and inclination, respectively).

Albert (2000), Seki, Miyoshi, Summers, and Meredith (2005), and Meredith et al. (2006) all used measurements from the Medium Electron Sensor A (MEA) instrument on the Combined Release and Radiation Effects Satellite (CRRES). CRRES operated in a Geostationary Transfer Orbit (GTO) orbit with launch parameters of 350 km x 5.3 R_E, 18.2° (perigee, apogee, and inclination, respectively).

Meredith et al. (2009) used measurements from the Proton/Electron Telescope (PET) instrument on the Solar Anomalous and Magnetospheric Particle Explorer (SAMPEX). SAMPEX operated in a Low Earth Orbit (LEO) with launch parameters of 512 km x 687 km, 81.7° (perigee, apogee, and inclination, respectively).

Benck, Mazzino, Cyamukungu, Cabrera, and Pierrard (2010) used measurements from both the Influence of Space Radiation on Advanced Components (ICARE) instrument on the Satelite de Aplicaciones Cientifico-C (SAC-C), and from the Instrument for Particle Detection (IDP) instrument on the Detection of Electro-Magnetic Emissions Transmitted from Earthquake Regions (DEMETER) satellite. SAC-C operated in a LEO orbit with launch parameters of 700 km x 700 km, 98.2° (perigee, apogee, and inclination, respectively). DEMETER operated in a LEO orbit with launch parameters of 710 km x 710 km, 98° (perigee, apogee, and inclination, respectively).

Su et al. (2012) used measurements from the SC3 instrument on the Spacecraft Charging At High Altitudes (SCATHA) satellite. SCATHA operated in a HEO orbit with launch parameters of 5.3 x 7.8 R_E, 7.7° (perigee, apogee, and inclination, respectively).

Text S2.

We now provide additional information on the automated algorithm that has been developed to identify exponential decays and calculate the e-folding times of the decays from the MagEIS electron measurements. We note that the specific MagEIS data used

are the same as in Claudepierre et al. (2019), which were provided as in that manuscript. The automated algorithm is based on the technique of Benck et al. (2010), which was in turn adapted from that of Meredith et al. (2006), but has been tailored somewhat to accommodate the unique features of the MagEIS data set. Our algorithm begins by considering a time series of electron flux in a fixed L and energy bin, which we denote (L,E). A smoothed version of the time series is separately calculated first to identify time intervals during which the smoothed flux is decreasing over some minimum time window length. This length is nominally 5 days, but larger values are used in some (L,E)-bins to obtain better fits and statistics - see below. We refer to the time interval over which the smoothed flux is decreasing as T, noting that it is at least 5 days long and can be longer if the smoothed flux is decreasing over a longer time scale. The length of the smoothing window is predetermined and varies in each (L,E)-bin, though it is nominally in the 1 (no smoothing) to 4 day range. Longer minimum time window lengths and longer smoothing windows are generally used in the inner zone, where the fluxes are less dynamic and more subject to fluctuations related to low counts (Poisson noise) and/or orbital effects. Data gaps in the smoothed time series shorter than 3 days are linearly interpolated across.

Once such an interval, T, has been identified, the original (non-smoothed) time series is analyzed and the smoothed time series is no longer used. The flux time series is fit with an exponential function, $J(t) = J_0 \exp(-t/\tau)$, over T. In order to construct a fit, we require that 90% of the flux values in T be non-zero and valid (e.g., non-fill value); there is no interpolation done on the original (non-smoothed) time series. Two goodness-of-fit

parameters relating the fit to the flux are recorded, the linear correlation coefficient and the percent error, which is defined as the median symmetric accuracy (Morley, Brito, and Welling (2018)). If the linear correlation coefficient, r, is greater than some predetermined threshold and the percent error is less than 25%, then the fit is accepted. As with the smoothing window and minimum time window lengths, the specific r-value for each (L,E)-bin is predetermined, with larger values (≥ 0.94) used at higher L and $r \geq 0.8$ almost everywhere (see below). We note, however, that the percent error threshold is fixed at 25% for all (L,E)-bins. For all subintervals of T that satisfy the minimum time window length requirement for the specific (L,E)-bin and the 90% requirement for valid flux values, additional exponential fits are obtained as described above. From all of the accepted fits within T, the fit with the largest r-value is retained as the single fit for the time interval T. The above process is then repeated on the next time interval over which the smoothed flux is decreasing, and so on. We emphasize that the smoothed time series is only used to identify time intervals during which the fluxes are decreasing; the exponential fits and statistical database are constructed using the original, daily-averaged flux time series. The entire process is then repeated in each (L,E)-bin to obtain a statistical database of decay intervals and decay timescales as a function of L and energy.

Figure S1 provides an example of the application of the automated procedure in four L bins and in the fixed energy bin of 467 keV. The daily averaged fluxes are shown in the middle (color) panel across the entire L and time range considered here, 01 Apr 2013 to 01 Apr 2018. The four line plot panels show flux profiles at the four indicated L

values. We see that the fluxes in the inner zone decay over long time scales ($\tau > 100$ d), while much more rapid decays are observed in the slot and outer zone ($\tau \sim 1-5$ d). The data gap at $L > 3$ early in the time interval is due to the operation of the MagEIS instrument in a mode that does not allow for background corrected data to be produced (see Claudepierre et al., 2015). While it may be tempting to use uncorrected data in such instances, we demonstrate in the main manuscript that background contamination can have a significant impact on lifetime calculations and lead to erroneous results.

As noted in the main manuscript, there are three primary parameters used in the algorithm to identify and accept exponential fits as valid. The first is the number of days used to smooth the daily-averaged flux time series, N_{smooth}. This parameter is shown in panel (a) Figure S2 and is used only to search for intervals when the flux is generally decreasing. Above L of 2.5, this parameter is generally in the 1-4 day range, but longer smoothing windows are used in the inner zone. This is because in the inner zone the daily-averaged fluxes are more sensitive to the orbital motion of the Van Allen Probes and its phasing with respect the geomagnetic equator (where a roughly 3 day periodicity is noted). Also, the decay timescales are much longer in the inner zone than elsewhere, so that smoothing over a longer time window helps identify longer time intervals from which the exponential fits are obtained.

Panel (b) in Figure S2 shows the minimum number of days required for a valid fit, N_{fit}, in each energy and L bin. Again, because the lifetimes in the inner zone are generally

longer, we require this parameter to be longer than elsewhere to provide a more accurate estimate of the decay timescale. Finally, panel (c) shows the threshold (minimum) linear correlation coefficient used to accept a fit as valid, r_{thresh}. This value is reduced in the inner zone below a nominal value of 0.9 used elsewhere so that more decay intervals can be identified. Because of the long decay timescales and the relative paucity of injections into the inner zone at higher energies, there are few events that satisfy the stringent criteria used in the outer slot and outer zone. In general, the criteria on the quality of the fits must be relaxed in the inner zone and at the highest energies to boost statistics.

It is worth noting that the automated algorithm described here is tailored to identify decay intervals on differential fluxes; it will not accurately compute the decay rates during a time interval that contains a two-stage decay in differential flux. This is because the algorithm will identify such an interval as a single decay interval and calculate the decay rate from whichever portion of the decay best satisfies the goodness-of-fit criteria. For a given decay interval this could, in principle, be either the rapid initial decay, or the slower more gradual decay that follows, so that any statistical database obtained would thus contain a mixture of both timescales present in two-stage decays. It is for these reasons that we do not compute electron lifetimes from integral fluxes in this work, nor compare such estimates with those obtained from other observational studies. A different algorithm than the one described here would be required to properly analyze decays in integral flux measurements.

Text S3.

The empirical decay timescale estimates from prior published works that appear in Table S1 were obtained following an exhaustive literature search. These represent, to the best of our knowledge, a complete set of prior decay timescale estimates with which we can compare our MagEIS estimates. We searched for decay timescale estimates that were made (i) in similar differential energy channels within the MagEIS energy range (30 keV - 4 MeV) and (ii) over a similar L range ($L = 1.3 - 6.0$). We did not require that the prior estimates be from a near-equatorial satellite or that a similar portion of the pitch-angle distribution was analyzed, as these criteria would have been much too restrictive. The only other estimates from a near-equatorial satellite and/or the same range of equatorial pitch angles are those from CRRES. For example, the flux data used to obtain the estimates in West et al. (1981) and Vampola (1971) are transformed to the magnetic equator or a reference pitch angle, which requires various assumptions on the angular distribution (e.g., Vampola (1971) extrapolates the high latitude fluxes in B/B_0 to obtain the equatorial flux). We also note that many of the prior estimates were obtained from comparatively less data than the 5 year database we have obtained (e.g., West et al. (1981) is from only two months of data, when only a handful of decays are observed at a given L and energy).

From this exhaustive literature search of prior decay time estimates, we note two important facts about our estimates obtained from MagEIS. First, they represent the first accurate empirical decay time estimates for electrons in the inner zone ($L < 2$) in differ-

ential energy channels over the 30 keV - 1 MeV energy range, aside from those of Benck et al. (2010). We note that the Benck et al. (2010) estimates are only available down to $L \approx 1.7$ and only in certain energy channels, and we have shown in the main manuscript that these estimates from LEO are out of family with the others and may be subject to instrumental effects. Note in particular that our estimates from MagEIS are the only ones available below $L = 2$ for energies less than 200 keV. Figure S3 demonstrates all of this explicitly. We note that there were several estimates of inner zone electron lifetimes made following high-altitude nuclear detonations in the late 1950s and early 1960s (e.g., Roberts, 1969, and references therein). However, those and other low L estimates (e.g., Rosen & Sanders, 1971) were all obtained using integral sensors (e.g., those that measure the flux above some threshold energy; see Figure 12 of West et al. (1981)), rather than the highly-resolved differential channels used here, and we demonstrate in the main manuscript that lifetimes calculated from integral sensors are difficult to properly interpret and can be misleading. For example, we note that Abel and Thorne (1998) compare their theoretical lifetimes at a fixed (differential) energy (0.5 MeV) with empirical estimates from an integral sensor (>0.5 MeV, their Figure 9). In our view, conclusively identifying the relevant scattering mechanism(s) at a given energy from such data/theory comparisons is highly uncertain since the theory is presented at a fixed energy while the observations mix decay rates across a wide range of energies. Similarly, decay timescale estimates obtained in the inner zone from the 2-6 MeV channel on SAMPEX (e.g., Baker et al., 2007; Meredith et al., 2009)) may be subject to similar decay rate mixing. In any event, there have not been observations of 2-6 MeV electrons at $L < 2.5$ during the Van Allen Probes era and thus

nothing available for us to compare with the SAMPEX estimates. Moreover, Selesnick (2015) has demonstrated the contamination of SAMPEX measurements by lower energy electrons, which may impact previously generated lifetimes from SAMPEX. Finally, we note that some estimates of electron decay timescales are available in the very inner portion of the inner zone (e.g., $L < 1.3$; Imhof and Smith (1965); Imhof, Reagan, and Smith (1967)), but these are below the L region considered in our work ($L > 1.3$).

Second, our MagEIS results represent the first accurate empirical decay time estimates for electrons throughout the radiation belts ($L = 1.3$ - 6.0) for differential energy channels <200 keV, aside from the two instances of West et al. (1981) (at 75 and 169 keV), the one instance of Benck et al. (2010) (at 169 keV), and those from Su et al. (2012), which are only available at $L > 5.5$. Figure S4 demonstrates this explicitly.

In the main manuscript, discrepancies were found between the MagEIS estimates and those obtained in prior works. In addition to the instrumental effects discussed there, there are a number of other factors that could lead to such discrepancies, such as differences in the techniques used to identify decay intervals and calculate lifetimes, orbital differences, solar cycle differences, and/or differences in the portions of the pitch angle distributions from which the lifetime estimates are made. Such differences have been noted by previous authors conducting similar analyses (e.g., Ripoll et al., 2015). These are not explored further in any detail here however, as we find the overall agreement between the prior estimates and those obtained from MagEIS to be quite good and argue that

the remaining differences are likely instrumental in nature. The fact that the majority of the discrepancies lie in a fixed L region ($L = 3$ - 5) also hints that high-energy electron contamination may be responsible, as one would expect the other potential factors to be systematic across all L.

In summary, we emphasize that prior to the Van Allen Probes mission, there was essentially no work on inner zone electrons for many years. With the high-quality data now available from the Van Allen Probes, there has been a renaissance in the study of the inner belt (e.g., Claudepierre et al., 2019, and references therein). The accurate empirical lifetime estimates in the inner zone obtained here facilitates and further enables the scientific study of inner zone electron dynamics by providing a critical parameter (the decay time) used in such studies (e.g., O'Brien et al., 2016). Similarly, by providing the first decay timescales for <200 keV electrons throughout the belts ($L = 1.3$ - 6.0), we expand the range of 1st invariants that may treated theoretically from the source and loss perspective.

References

Abel, B., & Thorne, R. M. (1998, February). Electron scattering loss in Earth's inner magnetosphere 1. Dominant physical processes. *J. Geophys. Res.*, *103*, 2385-2396. doi: 10.1029/97JA02919

Albert, J. M. (2000, Jan). Pitch Angle Diffusion as Seen by CRRES. *Adv. Space Res.*, *25*(12), 2343-2346. doi: 10.1016/S0273-1177(99)00520-7

Baker, D. N., Kanekal, S. G., Horne, R. B., Meredith, N. P., & Glauert, S. A. (2007, Oct).

Low-altitude measurements of 2-6 MeV electron trapping lifetimes at $1.5 \leq L \leq 2.5$. *Geophys. Res. Lett.*, *34*(20), L20110. doi: 10.1029/2007GL031007

Benck, S., Mazzino, L., Cyamukungu, M., Cabrera, J., & Pierrard, V. (2010, Mar). Low altitude energetic electron lifetimes after enhanced magnetic activity as deduced from SAC-C and DEMETER data. *Ann. Geophys.*, *28*(3), 849-859. doi: 10.5194/angeo-28-849-2010

Claudepierre, S. G., O'Brien, T. P., Blake, J. B., Fennell, J. F., Roeder, J. L., Clemmons, J. H., ... Larsen, B. A. (2015, July). A background correction algorithm for Van Allen Probes MagEIS electron flux measurements. *J. Geophys. Res.*, *120*, 5703-5727. doi: 10.1002/2015JA021171

Claudepierre, S. G., O'Brien, T. P., Looper, M. D., Blake, J. B., Fennell, J. F., Roeder, J. L., ... Spence, H. E. (2019, February). A Revised Look at Relativistic Electrons in the Earth's Inner Radiation Zone and Slot Region. *J. Geophys. Res.*, *124*, 934-951. doi: 10.1029/2018JA026349

Imhof, W. L., Reagan, J. B., & Smith, R. V. (1967). Long-term study of electrons trapped on low l shells. *J. Geophys. Res.*, *72*(9), 2371-2377. doi: 10.1029/JZ072i009p02371

Imhof, W. L., & Smith, R. V. (1965, May). Observation of nearly monoenergetic high-energy electrons in the inner radiation belt. *Phys. Rev. Lett.*, *14*, 885–887. doi: 10.1103/PhysRevLett.14.885

Meredith, N. P., Horne, R. B., Glauert, S. A., Baker, D. N., Kanekal, S. G., & Albert, J. M. (2009, Mar). Relativistic electron loss timescales in the slot region. *J. Geophys. Res.*, *114*(A3), A03222. doi: 10.1029/2008JA013889

Meredith, N. P., Horne, R. B., Glauert, S. A., Thorne, R. M., Summers, D., Albert, J. M., & Anderson, R. R. (2006, May). Energetic outer zone electron loss timescales during low geomagnetic activity. *J. Geophys. Res.*, *111*(A5), A05212. doi: 10.1029/2005JA011516

Morley, S. K., Brito, T. V., & Welling, D. T. (2018, January). Measures of Model Performance Based On the Log Accuracy Ratio. *Space Weather*, *16*, 69-88. doi: 10.1002/2017SW001669

O'Brien, T. P., Claudepierre, S. G., Guild, T. B., Fennell, J. F., Turner, D. L., Blake, J. B., ... Roeder, J. L. (2016, July). Inner zone and slot electron radial diffusion revisited. *Geophys. Res. Lett.*, *43*, 7301-7310. doi: 10.1002/2016GL069749

Ripoll, J. F., Chen, Y., Fennell, J. F., & Friedel, R. H. W. (2015, Jan). On long decays of electrons in the vicinity of the slot region observed by HEO3. *J. Geophys. Res.*, *120*(1), 460-478. doi: 10.1002/2014JA020449

Roberts, C. S. (1969, Jan). Pitch-angle diffusion of electrons in the magnetosphere. *Rev. Geophys.*, *7*, 305-337. doi: 10.1029/RG007i001p00305

Rosen, A., & Sanders, N. L. (1971). Loss and replenishment of electrons in the inner radiation zone during 1965-1967. *J. Geophys. Res.*, *76*, 110. doi: 10.1029/JA076i001p00110

Seki, K., Miyoshi, Y., Summers, D., & Meredith, N. P. (2005, Feb). Comparative study of outer-zone relativistic electrons observed by Akebono and CRRES. *J. Geophys. Res.*, *110*(A2), A02203. doi: 10.1029/2004JA010655

Selesnick, R. S. (2015, April). High-energy radiation belt electrons from CRAND. *J.*

Geophys. Res., *120*, 2912-2917. doi: 10.1002/2014JA020963

Su, Y.-J., Johnston, W. R., Albert, J. M., Ginet, G. P., Starks, M. J., & Roth, C. J. (2012, Aug). SCATHA measurements of electron decay times at $5 < L \leq 8$. *J. Geophys. Res.*, *117*(A8), A08212. doi: 10.1029/2012JA017685

Vampola, A. L. (1971). Natural variations in the geomagnetically trapped electron population. In E. A. Warman (Ed.), *Proceedings of the National Symposium on Natural and Manmade Radiation in Space* (p. 539-547).

West, J., H. I., Buck, R. M., & Davidson, G. T. (1981, Apr). The dynamics of energetic electrons in the earth's outer radiation belt during 1968 as observed by the Lawrence Livermore National Laboratory's spectrometer on Ogo 5. *J. Geophys. Res.*, *86*(A4), 2111-2142. doi: 10.1029/JA086iA04p02111

Table S1. Previous Empirical Estimates of Electron Decay Timescales

Reference	Spacecraft/Instrument	Orbit	Energy Channels[a]
Vampola (1971)	OV3-3/MES	HEO	475 & 957 keV
West et al. (1981)	OGO5/EPS	HEO	79-2830 keV (7 bins)
Albert (2000)	CRRES/MEA	GTO	510 keV
Seki et al. (2005)	CRRES/MEA	GTO	976 & 1582 keV
Meredith et al. (2006)	CRRES/MEA	GTO	214-1090 keV (8 bins)
Meredith et al. (2009)	SAMPEX/PET	LEO	2-6 MeV (1 bin)
Benck et al. (2010)	SAC-C/ICARE & DEMETER/IDP	LEO	160-1360 keV (13 bins)
Su et al. (2012)	SCATHA/SC3	HEO	57-289 keV (12 bins)

[a]Available for the comparisons; not necessarily representative of the full energy range of the instrument.

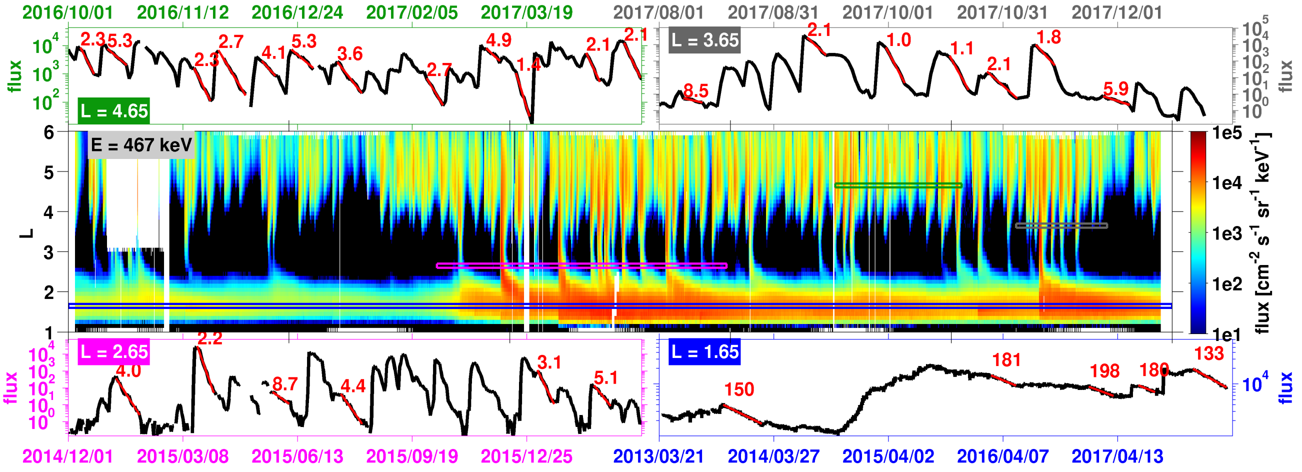

Figure S1. Daily-averaged, differential flux for 467 keV electrons averaged over equatorial pitch angles between 70° and 110°. The middle (color) panel shows the fluxes in L-versus-time format over the time interval 01 Apr 2013 to 01 Apr 2018. The four line plot panels show flux profiles at the four indicated L values; the time and L regions displayed are highlighted by colored boxes in the middle panel. Exponential decays identified by the automated algorithm are highlighted in red with the calculated decay (e-folding) times indicated, in days.

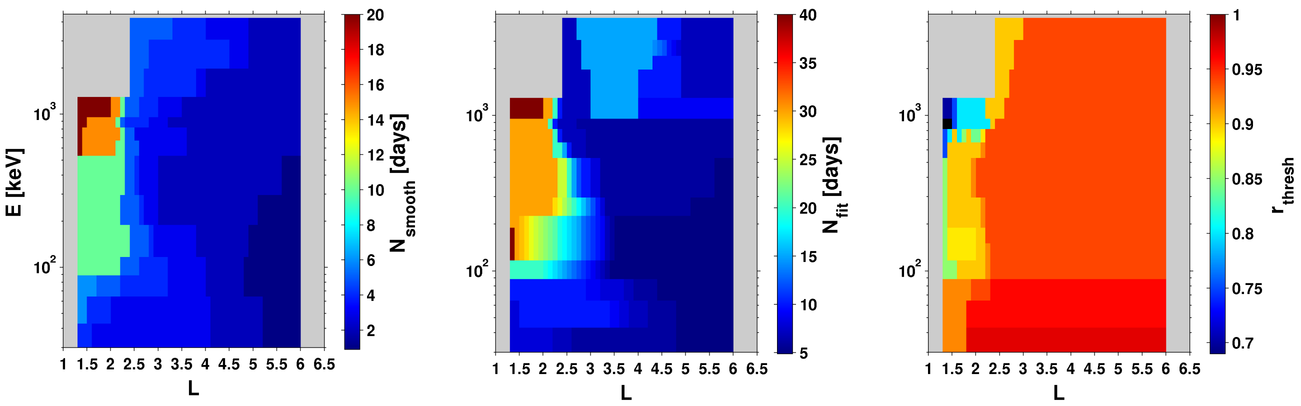

Figure S2. Overview of the three parameters used in the automated algorithm in each energy and L bin. **(a)** The number of days used to smooth the time series. **(b)** The minimum number of days required for a valid fit. **(c)** The threshold linear correlation coefficient used to accept a fit as valid.

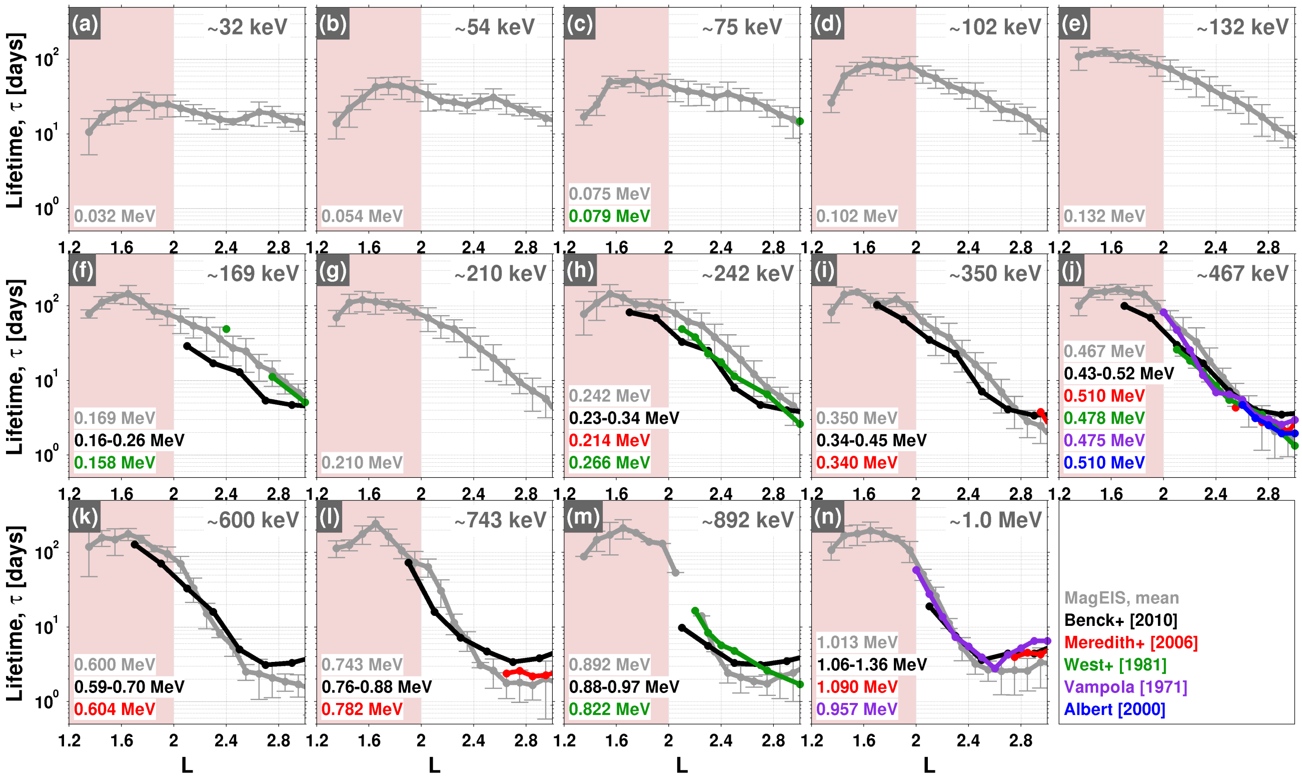

Figure S3. All available empirical lifetime estimates at $L < 3$ for differential energy channels between 30 keV and 1 MeV. The $L < 2$ region is highlighted to emphasize the lack of reliable decay timescale estimates available in this region, aside from our estimates from MagEIS.

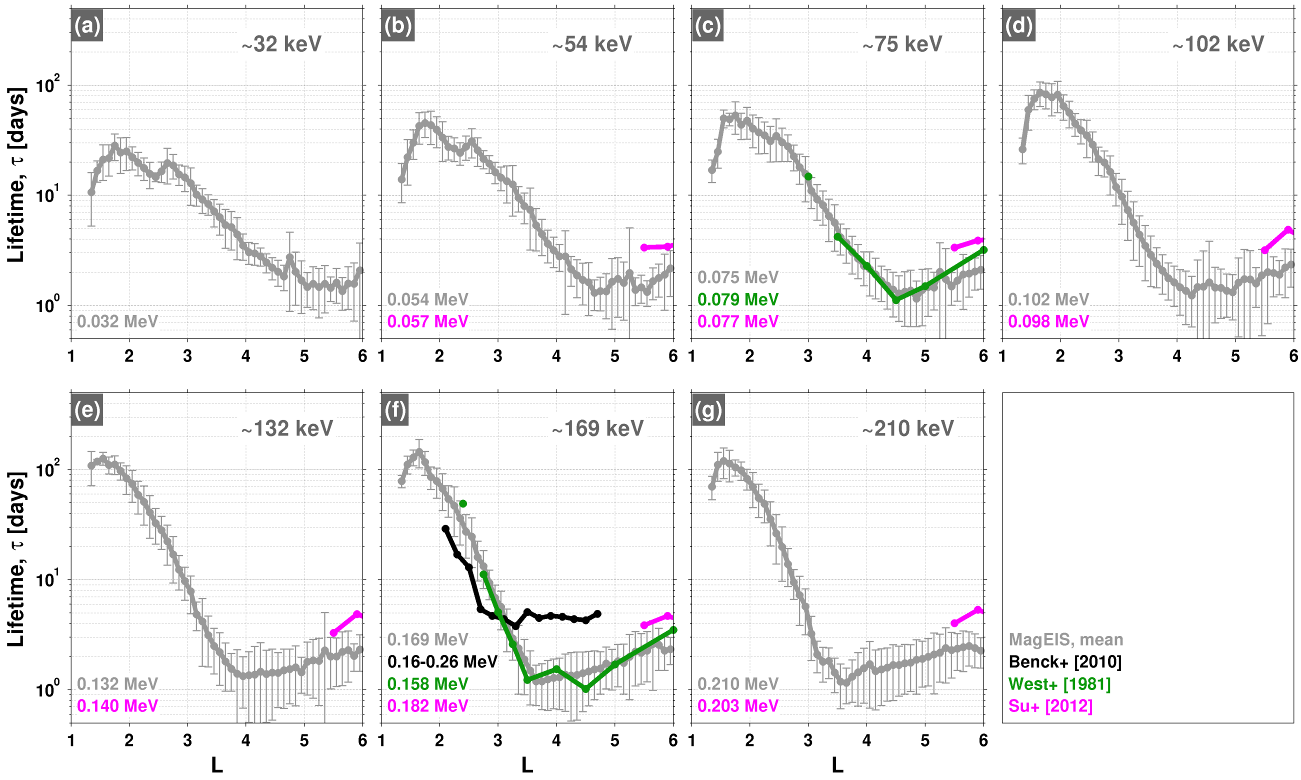

Figure S4. All available empirical lifetime estimates for differential energy channels between 30 keV and 210 keV across the entire $L = 1.3 - 6.0$ region. We emphasize the paucity of reliable decay timescale estimates available at these energies, aside from our estimates from MagEIS.
